# PrEdictive value of coMbined pre-test proBability and blOod gas anaLysis In pulmonary emboliSM—the EMBOLISM study

**DOI:** 10.1007/s11739-022-03075-w

**Published:** 2022-08-17

**Authors:** Moritz Meusel, Toni Pätz, Kim Gruber, Sebastian Kupp, Philipp-Johannes Jensch, Roza Saraei, Alexander Fürschke, Friedhelm Sayk, Ingo Eitel, Sebastian Wolfrum

**Affiliations:** 1 Department of Cardiology, Angiology and Intensive Care Medicine, University Heart Center Lübeck, University Hospital Schleswig-Holstein, University of Lübeck, German Center for Cardiovascular Research (DZHK), Partner site Hamburg/Kiel/Lübeck, Ratzeburger Allee 160, 23538 Lübeck, Germany; 2 Department of Radiology and Nuclear Medicine, University Hospital of Schleswig Holstein, Campus Lübeck, Ratzeburger Allee 160, 23538 Lübeck, Germany; 3 Department of Internal Medicine I, University of Lübeck, Ratzeburger Allee 160, 23538 Lübeck, Germany; 4 Emergency Department, University Hospital Schleswig-Holstein, Campus Lübeck, Ratzeburger Allee 160, 23538 Lübeck, Germany

**Keywords:** Pulmonary embolism, Pre-test probability, D-dimer, Blood gas analysis, Wells score

## Abstract

In patients with suspected pulmonary embolism (PE), the number of unnecessary computed tomography pulmonary angiography (CTPA) scans remains high, especially in patients with low pre-test probability (PTP). So far, no study showed any additional benefit of capillary blood gas analysis (BGA) in diagnostic algorithms for PE. In this retrospective analysis of patients with suspected PE and subsequent CTPA, clinical data, D-dimer levels and BGA parameters (including standardized PaO2) were analyzed. Logistic regression analyses were performed to identify independent predictors for PE and reduce unnecessary CTPA examinations in patients with low PTP according to Wells score. Of 1538 patients, PE was diagnosed in 433 patients (28.2%). The original Wells score (odds ratio: 1.381 [95% CI 1.300–1.467], *p* < 0.001) and standardized PaO2 (odds ratio: 0.987 [95% CI 0.978–0.996], *p* = 0.005) were independent predictors for PE. After cohort adjustment for low PTP a D-dimer cut-off < 1.5 mg/L (278 patients (18.1%) with 18 PE (6.5%)) was identified in which a standardized PaO2 > 65 mmHg reduced the number of unnecessary CTPA by 31.9% with a 100% sensitivity. This approach was further validated in additional 53 patients with low PTP. In this validation group CTPA examinations were reduced by 32.7%. No patient with PE was missed. With our novel algorithm combining BGA testing with low PTP according to Wells score, we were able to increase the D-Dimer threshold to 1.5 mg/L and reduce CTPA examinations by approximately 32%.

## Introduction

The clinical diagnosis of pulmonary embolism (PE) remains challenging. Current guidelines of the European Society of Cardiology (ESC) recommend the assessment of clinical pre-test probability (PTP) in hemodynamic stable patients with suspected PE [1]. The most frequently applied prediction rules are the Wells score for PE and the revised Geneva score. Accordingly, PE can be expected in approximately 12% in the “PE unlikely” category and 30% in the “PE likely” category, when the two-level classification is used [2]. In the “PE unlikely” category the subsequent laboratory testing for D-dimer is recommended and of major clinical importance. Normal D-dimer (< 0.5 mg/L) and/or negative age-adjusted D-dimer (age × 0.01 mg/L for patients older than 50 years) in the “PE unlikely” group safely rule out PE and effectively reduce unnecessary chest imaging [3–5].

Due to the low specificity of D-dimer, a positive D-dimer test in patients with a low PTP for PE is the major trigger for over testing with computed tomography pulmonary angiography (CTPA) [6]. Hence, even when adhering to the recommended algorithm, a low PTP and positive D-Dimer testing results in negative CTPA in up to 95% [7–10]. A viable approach to reduce the overuse of chest imaging via CTPA is the correction of the D-dimer cut-off. As investigated in the recent YEARS and PEGeD study, a D-dimer cut-off of less than 1.0 mg/L is safely applicable in the low-risk category [11, 12] and the usage of both scores resulted in a significant reduction of chest imaging by CTPA.

Next to assessment of PTP and, if necessary, D-dimer testing, capillary blood gas analysis (BGA) is regularly performed in patients with suspected PE. Commonly, hypoxemia and hypocapnia can be found in patients with PE [1]. Despite the pathophysiological plausibility of these findings, so far, no study was able to demonstrate an additional benefit of BGA testing to rule out PE [13, 14].

The aim of the present study was to reevaluate the role of BGA testing and its diagnostic benefits in patients with suspected PE, especially in patients with a low PTP. Using the standardized arterial oxygen tension (PaO2stand), we accounted for hyperventilation and hypocapnia, since both conditions affect the arterial oxygen tension (PaO2) [15] and are frequently seen in patients with PE [1]. In a novel approach, we combined the PTP and D-dimer with BGA results to reduce the need for unnecessary chest imaging in patients with suspected PE. We therefore analyzed the data of patients with suspected PE who were admitted at the emergency department (ED) of a large tertiary care university hospital and received chest imaging by CTPA.

## Materials and methods

### Study design and patients

The study was designed as a retrospective single center study and approved by the local ethics committee at the university of Lübeck (file number: 19-318A). The study cohort was derived from the ED of the university hospital of Schleswig–Holstein in Lübeck, a tertiary hospital with over 40.000 patient contacts per year. Data archive of electronic patient records were searched for CTPA to verify the suspected diagnosis of PE from 07/2015 to 06/2020. The hospital´s standard operating procedure for patients with suspected PE includes the assessment of PTP using Wells score and, if necessary, D-dimer testing (D-dimer Hemosil HS 500 with the ACL Analyzer Top 750, Werfen, Germany). Age-adjusted cut-off values in patients were used in patients over 50 years of age, otherwise a cut-off of 0.5 mg/L was applied. Therefore, the present study cohort represents a preselected patient cohort in which a CTPA has been considered necessary to verify or safely rule out PE according to the current ESC guideline recommendations [1]. In addition, data of the original and revised Geneva score were collected retrospectively.

Patient records of each patient were searched for relevant parameters. Using common BGA parameters, we standardized the arterial oxygen tension (PaO2) to arterial carbon dioxide tension (PaCO2) to account for hyperventilation and hypocapnia, since both conditions affect the PaO2 [15]. This correction resulted in a standardized PaO2 (PaO2stand = PaO2 – 1.66 × (40-PaCO2)). This standardized PaO2 was retrospectively calculated. All patients were classified into the “PE unlikely” (≤ 4 points) and the “PE likely” group (> 4 points) according to Wells criteria [12, 16, 17]. To compare the results of our novel BGA-based algorithm with the PEGeD algorithm [12], a direct comparison was carried out within the group of patients with low PTP.

### Statistical analysis

The statistical analysis was performed with IBM SPSS Statistics 26.0 with a two-sided *p* value of < 0.05 considered statistically significant. Categorical variables were analyzed using Chi^2^ test or Fisher's exact test and are expressed as numbers and percentages. Continuous variables were investigated using Mann–Whitney-U test and are expressed as median and interquartile range (IQR). Baseline characteristics and BGA parameters were compared between patients with confirmed PE and patients without PE. Independent predictors of PE were analyzed using logistic regression analysis and presented as odds ratios (OR) within 95% confidence intervals (CI). Moreover, to analyze the predictive value of the significant parameters in the first step, we used a stepwise forward regression to evaluate the independent impact of these parameters on the presence of PE. For missing data no imputation was performed.

### CT pulmonary angiography

The CTPA was performed by a 128 detector Siemens Somatom Definition AS + and AS scanner (Siemens Medical System). According to hospital standard operating procedures the CTPA was performed in a lying supine position. During the procedure, patients were asked to hold their breath; if this was not possible, the patients were allowed to continue breathing shallowly. Scan volume included in its cranio-caudal direction the clavicle and the diaphragm. Detector scan area was up to 800 mm. The slice thickness was 1 mm and the tube current and voltage was 100mAs as quality reference using CARE dose and 120 kV. Low osmolar nonionic contrast medium (100 ml) was administered through a permanent venous catheter at a flow rate of 5 ml/s.

## Results

### Baseline characteristics

A total of 1538 patients with suspected PE and subsequent CTPA were included in this study. Table 1 shows the detailed baseline characteristics and risk stratification of patients with and without PE. Overall, PE was ruled out in 71.8% (*n* = 1105) of all patients. Patients with confirmed PE (*n* = 433) had more frequently chest pain (*p* = 0.003), dyspnea (*p* < 0.001) and clinical signs of deep vein thrombosis (*p* < 0.001) but a lower rate of syncope (*p* = 0.039). In addition, typical ECG presentations like S1Q3 pattern (*p* = 0.002) and T wave inversion in V1–V4 (*p* < 0.001) were more frequently observed in patients with PE. Furthermore, patients with PE had a higher rate of a positive history of previous PE or deep vein thrombosis (*p* < 0.001) as well as oxygen administration at hospital admission (*p* < 0.001). Regarding blood tests, patients with PE had an elevated rate of positive Troponin test (*p* = 0.001) and a higher D-dimer serum concentration (*p* < 0.001). An increased frequency of right heart dysfunction in CTPA and/or echocardiography was also detected in patients with confirmed PE (*p* < 0.001).

**Table 1:** Baseline characteristics and risk stratification

	PE negative (*n* = 1105)	PE positive (*n* = 433)	*p* value
Male	558 (50.5)	204 (47.1)	*p* = 0.232
Age	69 (57, 78)	71 (57, 79)	*p* = 0.216
Central PE		204 (47.1)	
Segmental PE		183 (42.3)	
Subsegmental PE		46 (10.6)	
Chest pain	396 (35.8)	190 (43.9)	***p* = 0.003**
Dyspnea	664 (60.1)	320 (73.9)	***p* < 0.001**
Hemoptysis	47 (4.3)	16 (3.7)	*p* = 0.619
Syncope	179 (16.2)	52 (12.0)	***p* = 0.039**
Clinical signs of deep vein thrombosis	124 (11.2)	117 (27.0)	***p* < 0.001**
Tachycardia (> 100 bpm)	315 (28.5)	144 (33.3)	*p* = 0.067
S1Q3 pattern	86 (7.8)	56 (12.9)	***p* = 0.002**
T wave inversion in V1-V4	62 (5.6)	63 (14.5)	***p* < 0.001**
Complete/incomplete RBBB	121 (11.0)	62 (14.3)	*p* = 0.067
History of cardiovascular disease	263 (23.8)	90 (20.8)	*p* = 0.206
History of pulmonary disease	267 (24.2)	88 (20.3)	*p* = 0.108
History of renal disease	106 (9.6)	32 (7.4)	*p* = 0.174
Active cancer	193 (17.5)	78 (18.0)	*p* = 0.800
Previous PE or DVT	100 (9.0)	114 (26.3)	***p* < 0.001**
D-dimer^a^, mg/L	1.67 (1.13, 3.66)	4.67 (2.13, 10.22)	***p* < 0.001**
Administration of oxygen^c^			***p* < 0.001**
Unknown	112 (10.1)	25 (5.8)	
Yes	170 (15.4)	108 (25)	
No	822 (74.5)	299 (69.2)	
Oxygen flow rate in liters per minute^d^	3 (2, 5.75)	4 (2, 6)	*p* = 0.227
Right heart dysfunction in CTPA or echocardiography^e^	89 (10.7)	182 (42.3)	***p* < 0.001**
Elevated Troponin serum concentration^f^	337 (53.0)	250 (63.1)	***p* = 0.001**
Elevated NTpro-BNP serum concentration^g^	185 (53.0)	119 (46.5)	*p* = 0.113
sPESI		1 (0, 1)	
EMR: low risk^h^		98 (22.7)	
EMR: intermediate low risk^h^		187 (43.3)	
EMR: intermediate high risk^h^		128 (29.6)	
EMR: high risk^h^		18 (4.2)	
Confirmed DVT^i^	18 (18.0)	226 (55.8)	***p* < 0.001**

Data presented as n/N (%) or median (IQR). Numbers in bold type indicate a significant difference

*PE* pulmonary embolism, *DVT* deep vein thrombosis, *RBBB* right bundle branch block, *BGA* blood gas analysis, *CTPA* computed tomography pulmonary angiography, *sPESI* simplified PESI (Pulmonary Embolism Severity Index), *EMR* early mortality risk of in-hospital or 30-day death

^a^*n* = 1419, ^b^*n* = 1105, ^c^*n* = 1536, ^d^*n* = 265, ^e^*n* = 1265, ^f^*n* = 1032, ^g^*n* = 605, ^h^*n* = 431, ^i^*n* = 505, ^j^*n* = 437

### Blood gas analysis and pre-test probability

To investigate the diagnostic value of BGA measurements in suspected PE, all patients without BGA documentation were excluded (*n* = 7, 0.5%). BGA results were not available in 2 patients (0.5%) with confirmed PE and 5 patients (0.5%) without PE. Most of the BGA results were generated by capillary BGA (91.8% in PE-negative patients, 95.6% in PE-positive patients). Only a minority of all BGA results based on venous BGA (3.0% with PE, 1.8% without PE) or had an unknown extraction point of the BGA (4.7% with PE, 2.3% without PE). For further analysis we only considered patients with capillary BGA, since these have the highest validity for the oxygen tension. To eliminate the influence of oxygen administration, all patients with administered oxygen at hospital admission were excluded. Consequently, a total of 1073 patients were included in this analysis, the characteristics are presented in Table 2. All PTP scores were significantly higher in patients with confirmed PE (*p* < 0.001 for all scores) and although both patient groups showed a slightly alkaline pH, a statistically significant higher pH was observed in patients with PE (*p* = 0.007). Furthermore, a lower level PaO2 (*p* = 0.006) and PaO2stand (*p* < 0.001) could be detected in patients with confirmed PE.

**Table 2:** Blood gas analysis and pretest probability scores of patients with capillary BGA

	PE negative (*n* = 748)	PE positive (*n* = 291)	*p* value
Wells score	1.5 (0, 4)	4.5 (2.5, 6)	***p* < 0.001**
Simplified Wells score	1 (0, 2)	2 (1, 3)	***p* < 0.001**
Original revised Geneva score	5 (3, 6)	6 (4, 9)	***p* < 0.001**
Simplified revised Geneva score	2 (1, 3)	3 (2, 4)	***p* < 0.001**
BGA pH^a^	7.46 (7.43, 7.49)	7.47 (7.45, 7.50)	***p* = 0.007**
BGA PaCO2	33 (29, 37)	32 (29, 36)	*p* = 0.103
BGA PaO2	67 (58, 77.75)	65 (58, 73)	***p* = 0.006**
BGA HCO3-^b^	23.7 (21.5, 25.7)	23.6 (21.7, 25.9)	*p* = 0.995
BGA BE^c^	0.6 (– 1.0, 2,4)	0.8 (– 1.15, 2.5)	*p* = 0.590
BGA lactate^d^	1.1 (0.8, 1.6)	1.0 (0.8, 1.5)	*p* = 0.080
BGA saturation^e^	95.95 (93, 97.2)	95 (93, 97)	*p* = 0.082
PaO2stand	57.02 (47.06, 66.66)	53.36 (43.08, 61.54)	***p* < 0.001**

Data presented as median (IQR). Numbers in bold type indicate a significant difference

*PE* pulmonary embolism, *BGA* blood gas analysis, *PaCO2* arterial carbon dioxide tension, *PaO2* arterial oxygen tension, *HCO3-* bicarbonate, *BE* base excess. *PaO2stand* standardized to a PaCO2 of 40 mm Hg [15]

^a^*n* = 1072, ^b^*n* = 1051, ^c^*n* = 1056, ^d^*n* = 1060, ^e^*n* = 984

### Predictors of PE

Table 3 presents the results of the binary and multiple stepwise logistic regression analyses for the presence of PE. In binary logistic regression analysis of all BGA parameters and PTP scores, the original Wells score (*p* < 0.001), the simplified Wells score (*p* < 0.001), the original revised Geneva score, the simplified revised Geneva score (*p* < 0.001) as well as the PaO2 (*p* = 0.001) and the PaO2stand (*p* < 0.001) showed significant predictive associations with confirmed PE. In multiple logistic regression analysis including all significant parameters of simple binary regression only the original Wells score (odds ratio: 1.381 [95% CI 1.300–1.467], *p* < 0.001) and the PaO2stand (odds ratio: 0.987 [95% CI 0.978–0.996], *p* = 0.005) proved to be significant predictors for PE.

**Table 3:** Binary and multiple stepwise logistic regression analysis of baseline characteristics in patients with confirmed pulmonary embolism

	Binary logistic regression PE OR (95% CI)	Multiple logistic regression PE OR (95% CI)	*p* value Binary/multiple
Wells score	1.391 (1.310–1.477)	1.381 (1.300–1.467)	***p* < 0.001/*p* < 0.001**
Simplified Wells score	1.954 (1.711–2.231)		***p* < 0.001**
Original revised Geneva score	1.192 (1.142–1.244)		***p* < 0.001**
Simplified revised Geneva score	1.518 (1.352–1.705)		***p* < 0.001**
BGA pH^a^	8.238 (0.728–93.195)		*p* = 0.088
BGA PaCO2	0.984 (0.963–1.006)		*p* = 0.159
BGA PaO2	0.983 (0.974–0.993)		***p* = 0.001**
BGA HCO3-^b^	0.989 (0.951–1.028)		*p* = 0.580
BGA BE^c^	0.985 (0.949–1.022)		*p* = 0.414
BGA lactate^d^	0.877 (0.756–1.019)		*p* = 0.086
BGA saturation^e^	0.998 (0.967–1.031)		*p* = 0.916
PaO2stand	0.983 (0.974–0.983)	0.987 (0.978–0.996)	***p* < 0.001/*p* = 0.005**

Data presented as odds ratio (OR) with 95% confidence interval (CI). Numbers in bold type indicate a significant difference

*PE* pulmonary embolism, *BGA* blood gas analysis, *PaCO2* arterial carbon dioxide tension, *PaO2* arterial oxygen tension, *HCO3-* bicarbonate, *BE* base excess, *PaO2stand* standardized to a PaCO2 of 40 mmHg [15]

^a^*n* = 1072, ^b^*n* = 1051, ^c^*n* = 1056, ^d^*n* = 1060, ^e^*n* = 984

### BGA-optimized pre-test probability

**Fig. 1:**
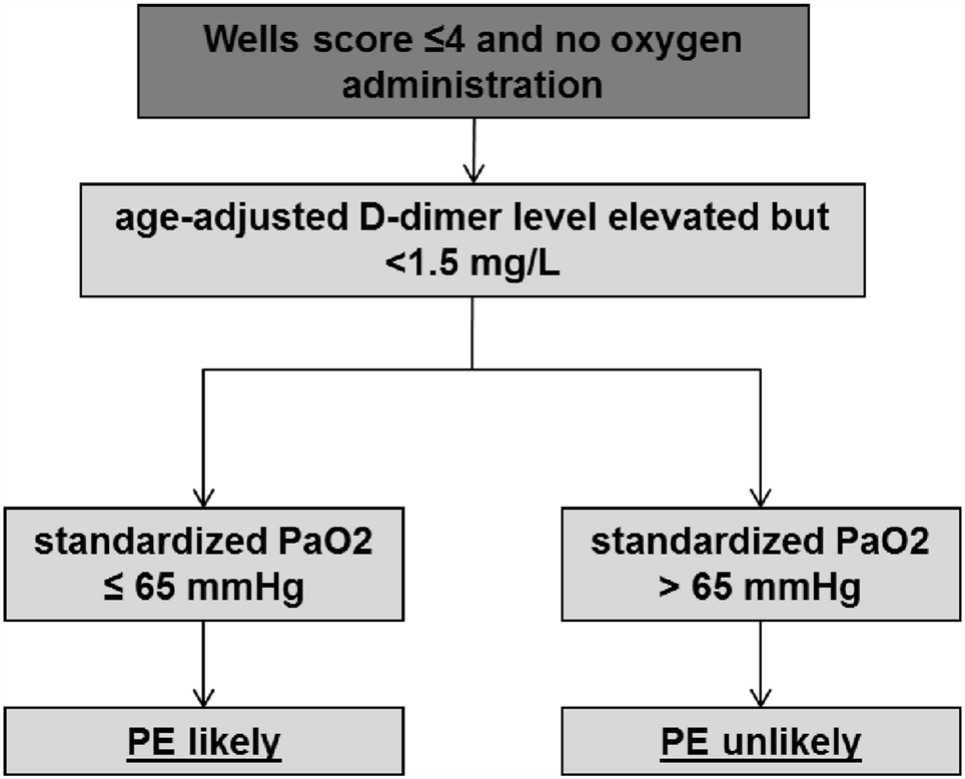
Workflow for patients with low pre-test probability and available capillary blood gas analysis. *PE* pulmonary embolism, standardized PaO2 partial pressure of oxygen standardized to a PaCO2 of 40 mmHg [15]

In a next step we tried to reduce the number of unnecessary CTPA examinations. Therefore, we used all significant parameters shown in Table 3. We focused on the group of patients with a low PTP, since the number of unnecessary CTPA tests is usually high in this cohort. For this approach, the cohort with a low PTP (defined with a Wells score ≤ 4), available BGA and age-adjusted increased D-dimers was identified. A total of 688 patients met these criteria and PE was detected in a total of 18.3% (*n* = 126) in this population. Although there was a significant difference between the standardized PaO2 values comparing patients with or without PE (without PE: median = 57.58 mmHg [95% CI 47.76–67.03]; with PE: median = 52.39 mmHg [95%CI 43.06–62.70]; *p* = 0.002), no cut-off value could be identified for which at least one patient with PE was not detected. Therefore D-dimer concentration were included into an exploratory analysis. The sensitivity of the standardized PaO2 could be increased significantly if the D-dimers within this cohort were < 1.5 mg/L. A total of 278 of 688 patients met these criteria and PE was detected in a total of 6.5% (*n* = 18) of these patients. Within this sub cohort, a standardized PaO2 > 65 mmHg could be identified as the cut-off value with a sensitivity of 100% (patients without PE: median = 57.71 mmHg vs. patients with PE: median = 50.74 mmHg). Through application of this variable, we were able to reduce the number of unnecessary CTPA test by 31.9% (83 of 260 patients without PE) without missing one patient with confirmed PE. Based on these results, we created the workflow as shown in Fig. 1.

### Validation group analysis and algorithm comparison

**Fig. 2:**
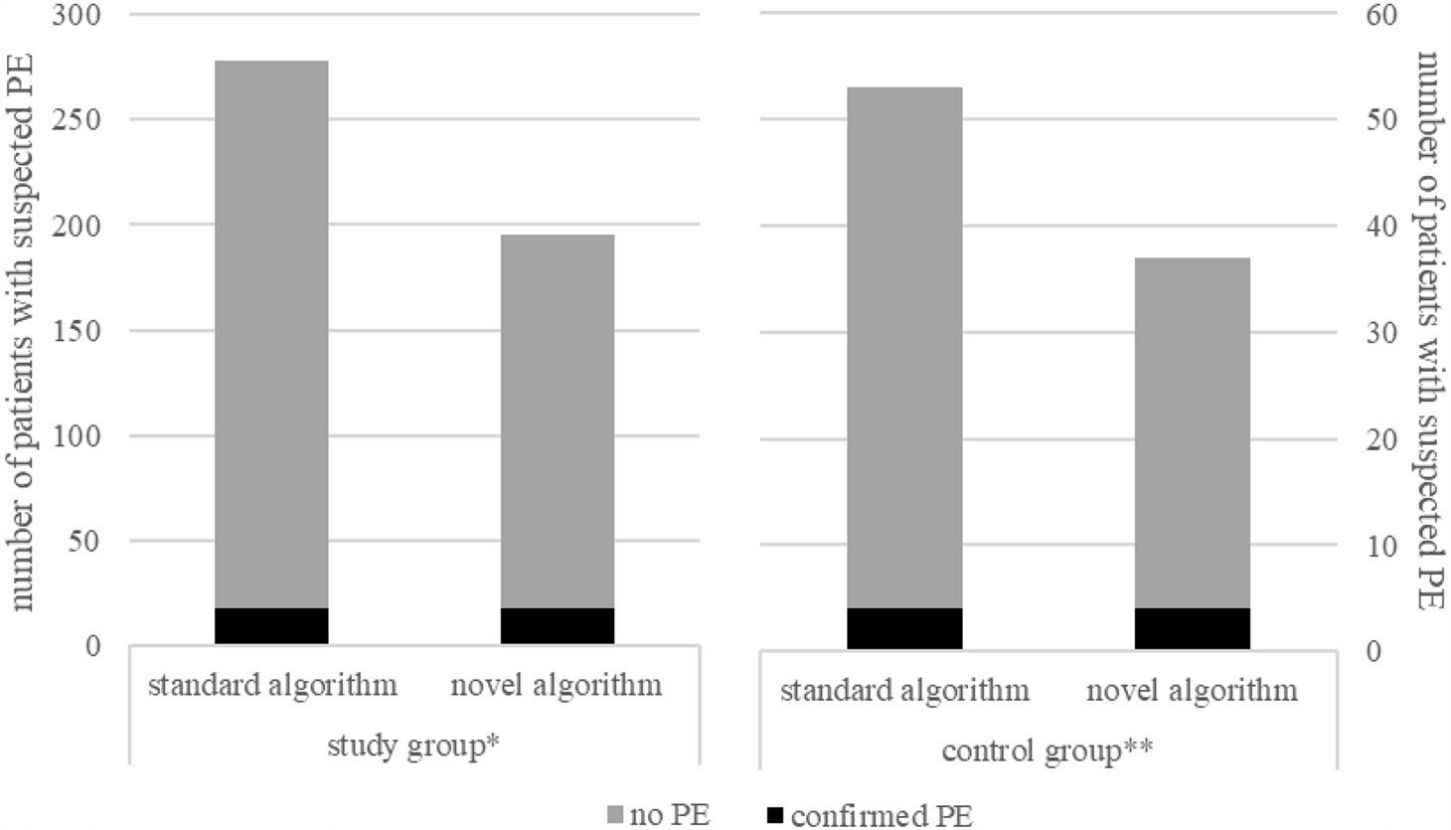
Number of examined patients with low pre-test probability analyzed separately according to standard algorithm and novel algorithm. *n = 278, **n = 53. *PE* pulmonary embolism

From 07/2020 to 03/2021, a total of 53 patients met the criteria of our algorithm (low PTP, D-dimer < 1.5 mg/L and capillary BGA, no oxygen administration). All these patients were included in our control group and PE was present in 7.6% (4 of 53 patients). With application of our algorithm, we again did not miss a single patient with PE and the number of unnecessary CTPA examinations could be reduced by 32.7% (16 of 49 patients without PE, Fig. 2).

Moreover, we extracted all patients of our primary cohort with a low PTP according to the original Wells Score and BGA (*n* = 688) and analyzed the subgroup of patients with a D-dimer above 1 mg/L (PEGeD cut-off, *n* = 590) to compare the effects of our novel algorithm to the PEGeD algorithm [12]. Within this subgroup, a total of 55 patients (9.3% of all 590 patients) had D-dimer values below 1.5 mg/L and a PaO2stand of > 65 mmHg. Thus, according to our algorithm, no CTPA would have been necessary, according to our algorithm. Within this group no patient with PE was missed.

## Discussion

To the best of our knowledge, this is the first investigation to demonstrate an additive value of BGA to reduce the number of unnecessary CTPA examinations in patients with suspected PE. In this retrospective study with a well-defined cohort of patients with suspected PE and subsequent CTPA, standardized PaO2 represents a useful tool to reduce unnecessary chest imaging in patients with low PTP. In the subgroup with a low PTP (Wells score ≤ 4 points), a standardized PaO2 of > 65 mmHg enabled us to increase the D-dimer threshold to < 1.5 mg/L to safely exclude PE. By application of our novel algorithm, a reduction in CTPA rate by approximately 32% could be achieved, without missing a single patient with PE, when age-adjusted D-dimer is considered the gold standard. The particular strength of this novel algorithm is that it is based on two components that are well known and frequently used in clinical practice. Furthermore, even when compared to the recently published PEGeD algorithm [12], the application of our novel algorithm using BGA parameters and a higher D-dimer cut-off levels in patients with low PTP the number of unnecessary CTPA can be reduced by 9.3% without missing a patient with PE. Our novel diagnostic algorithm should be applied to patients in the emergency department when pulmonary embolism is clinically suspected.

Current guidelines recommend the assessment of PE probability in hemodynamic stable patients by the Wells or revised Geneva score with subsequent D-dimer testing and CTPA, if necessary [1]. Therefore, the indication whether to perform CTPA or not in the “PE unlikely” group depends on D-dimer testing. Testing for D-dimer has a poor specificity for venous thromboembolism and elevated D-dimer levels can be found in numerous conditions such as malignant diseases, pregnancy, and infections [18, 19]. Thus, elevated D-dimer levels above 0.5 mg/L are frequent and a leading cause for chest imaging in the “PE unlikely” group. In up to 95% CTPA is negative for PE in this cohort [7–10, 20]. In 2014, Righini et al. reported that an adjustment of D-dimer levels to the patients’ age (age × 0.01 mg/L for patients older than 50 years) can safely exclude PE. The age-adjusted D-dimer levels increased the exclusion rate of PE from 6.4 to 30% [3] and reduced the unnecessary CTPA, accordingly. To further decrease CTPA frequency in suspected PE, studies then attempted to increase the D-dimer threshold. According to the YEARS algorithm, a D-dimer cut-off of < 1.0 mg/L is appropriate in patients with a low clinical PTP. The application of the YEARS algorithm avoided a total of 14% of CTPA when compared to standard care (Wells score and a fixed D-dimer cut-off of < 0.5 mg/L) [11]. Recently the PEGeD study confirmed the safety of a D-dimer cut-off of < 1.0 mg/L in patients with a low PTP, as assessed by the Wells score (≤ 4 points) [12]. So far, no study successfully investigated higher D-dimer cut-off levels than 1.0 mg/L. Our analysis included patients with suspected PE, clinical PTP as assessed by Wells score, age-adjusted D-dimer levels and CTPA. By adding the standardized PaO2 to a low PTP by Wells score (≤ 4 points) we were able to increase the D-dimer cut-off to < 1.5 mg/L in the “PE unlikely” group.

The diagnostic significance of BGA in the assessment of suspected PE has been controversially discussed in the literature. On the one hand, the assessment of PE probability by the original Geneva score includes blood gas parameters such as the PaO2 and PaCO2 [21]. Furthermore, in patients with malignant disease a benefit of BGA in the diagnosis of PE was recently demonstrated. In this study, the PaO2 was significantly lower in cancer patients and an alveolar-arterial gradient > 20 had 100% sensitivity and negative predictive value [22]. On the other hand, several studies investigating the usage of BGA parameters demonstrated no additional diagnostic benefit. Neither the alveolar–arterial oxygen tension gradient alone, nor a combination with a PaCO2 > 35–36 mmHg and/or the absence of prior thromboembolic disease reached a sensitivity of 100% and were able to increase the PTP in PE [13, 14, 23–25]. Since its widespread utilization and the common presence of hypoxemia and hypocapnia in patients with PE, BGA, especially the PaO2 and PaCO2, remains a simple bedside tool for clinical evaluation of suspected PE. The aim of the study was to use established standard diagnostics in combination with the BGA to optimize the indication for further chest imaging in the low PTP cohort without missing patients with PE. In our approach, we accounted for the pathophysiological changes commonly observed in PE, i.e., hyperventilation and hypocapnia, by application of the formula proposed by Mays et al. [15]. This correction of PaO2 resulted in a standardized PaO2. The standardized PaO2 is the first BGA parameter to show a sensitivity of 100% in excluding PE in our subgroup of low PTP and D-dimer cut-off < 1.5 mg/L.

The overall prevalence of PE in our registry was 28.2%, which is in accordance with the current literature [26, 27]. While this provides a certain comparability to other studies, it also shows the high proportion of patients with negative CTPA. In fact, there is evidence of an extensive overuse of CTPA in patients with suspected PE due to non-adherence to pre-test scoring algorithms [28, 29]. This aspect must be addressed by strict adherence to pre-test scores and other clinical tools such as D-dimer testing to reduce the number of unnecessary chest imaging and ionizing radiation exposure.

### Limitations

Several aspects might impair the interpretation of our results. First, this is a retrospective single center study based on hospital records. We used age-adjusted D-Dimer testing for a preselection. Therefore, the benefit of other algorithms like YEARS cannot be easily verified with our study. In addition, it must be considered that particularly the variable "alternative diagnosis is less likely than PE" depends on the clinical assessment by the physician in the ED. However, since 3 points are related to this variable, some patients may be classified into a higher PTP group and this in turn may have influenced the results. Finally, despite presenting a large and well characterized patient cohort with suspected PE larger studies are needed to prospectively confirm the value of our proposed score in suspected PE patients with low PTP.

## Conclusion

In this large cohort of patients with suspected PE and subsequent CTPA, we were able to show that BGA had an additional benefit to reduce unnecessary chest imaging in patients with low PTP.

## Abbreviations

BGA: Blood gas analysis
CI: 95% Confidence interval
CTPA: Computed tomography pulmonary angiography
DVT: Deep vein thrombosis
ECG: Electrocardiogram
ED: Emergency department
EMR: Early mortality risk of in-hospital or 30-day death
ESC: European Society of Cardiology
IQR: Interquartile range
OR: Odds ratios
PaCO2: Arterial carbon dioxide tension
PaO2: Arterial oxygen tension
PaO2stand: Standardized arterial oxygen tension
PTP: Pre-test probability
RBBB: Right bundle branch block
sPESI: Simplified PESI (Pulmonary Embolism Severity Index)
